# Genome-Wide Transcriptional Response of the Archaeon *Thermococcus gammatolerans* to Cadmium

**DOI:** 10.1371/journal.pone.0041935

**Published:** 2012-07-27

**Authors:** Arnaud Lagorce, Aude Fourçans, Murielle Dutertre, Brice Bouyssiere, Yvan Zivanovic, Fabrice Confalonieri

**Affiliations:** 1 Université Paris-Sud 11, UMR-CNRS 8621, Institut de Génétique et de Microbiologie, Laboratoire de Génomique des Archaea, Orsay, France; 2 Laboratoire de Chimie Analytique BioInorganique et Environnement, IPREM, UMR-CNRS 5254, Pau, France; University of Florida, United States of America

## Abstract

*Thermococcus gammatolerans*, the most radioresistant archaeon known to date, is an anaerobic and hyperthermophilic sulfur-reducing organism living in deep-sea hydrothermal vents. Knowledge of mechanisms underlying archaeal metal tolerance in such metal-rich ecosystem is still poorly documented. We showed that *T. gammatolerans* exhibits high resistance to cadmium (Cd), cobalt (Co) and zinc (Zn), a weaker tolerance to nickel (Ni), copper (Cu) and arsenate (AsO_4_) and that cells exposed to 1 mM Cd exhibit a cellular Cd concentration of 67 µM. A time-dependent transcriptomic analysis using microarrays was performed at a non-toxic (100 µM) and a toxic (1 mM) Cd dose. The reliability of microarray data was strengthened by real time RT-PCR validations. Altogether, 114 Cd responsive genes were revealed and a substantial subset of genes is related to metal homeostasis, drug detoxification, re-oxidization of cofactors and ATP production. This first genome-wide expression profiling study of archaeal cells challenged with Cd showed that *T. gammatolerans* withstands induced stress through pathways observed in both prokaryotes and eukaryotes but also through new and original strategies. *T. gammatolerans* cells challenged with 1 mM Cd basically promote: 1) the induction of several transporter/permease encoding genes, probably to detoxify the cell; 2) the upregulation of Fe transporters encoding genes to likely compensate Cd damages in iron-containing proteins; 3) the induction of membrane-bound hydrogenase (Mbh) and membrane-bound hydrogenlyase (Mhy2) subunits encoding genes involved in recycling reduced cofactors and/or in proton translocation for energy production. By contrast to other organisms, redox homeostasis genes appear constitutively expressed and only a few genes encoding DNA repair proteins are regulated. We compared the expression of 27 Cd responsive genes in other stress conditions (Zn, Ni, heat shock, γ-rays), and showed that the Cd transcriptional pattern is comparable to other metal stress transcriptional responses (Cd, Zn, Ni) but not to a general stress response.

## Introduction

While trace amounts of several metals such as iron (Fe), manganese (Mn), copper (Cu), nickel (Ni), cobalt (Co) and zinc (Zn) are essential to support cell growth, elevated amounts induce cell toxicity [1]. Other non-essential metals, such as cadmium (Cd), cause severe toxicity even at very low concentrations (reviewed in [2]). Cd induces several cellular dysfunctions and enhances mutagenesis and cell death. At the protein level, Cd ions can displace cognate metals leading to enzymatic inactivation and induce toxicity [3]. Cd also generates oxidative stress resulting in the production of reactive oxygen species (ROS; [3]). Thus, microbes have evolved to efficiently adjust cellular homeostasis by regulating metal import, and in the case of a massive exposure, activate efflux pumps [4]. A complementary mechanism developed by many organisms consists of metal chelation by cysteine-rich peptides such as metallothioneins, glutathione, phytochelatins, or with inorganic polyphosphate [2], [5], [6]. The complexes are sequestered into a limited area to prevent the free circulation of metal ions in the cytosol.

During the last decade, transcriptomic analyses performed in Bacteria and Eukarya to investigate Cd toxicity have provided results illustrating the involvement of similar or specific strategies to withstand Cd ions exposure. Human cells exhibit induction of genes encoding metallothioneins, anti-oxidant and heat shock proteins related to cellular protection and damage control mechanisms [7]. The immediate Cd response of *Arabidopsis thaliana* consists of an upregulation of genes involved in sulfur assimilation-reduction and glutathione metabolism in roots, while several genes involved in phenylpropanoid biosynthesis are induced in leaves [8]. Genome-wide expression patterns of *Saccharomyces cerevisiae* exhibit an upregulation of genes involved in glutathione synthesis and sulfur amino acid metabolism, coupled with an upregulation of common stress-response genes [9]. *Escherichia coli* cells downregulate protein biosynthesis, shift to an anaerobic metabolism and enhance several stress response systems [10]. Additionally, the cyanobacterium *Synechocystis* PCC6903 produces and repairs Fe-requiring metalloenzymes by a moderate increase of Fe uptake and breakdown of the Fe-rich photosynthesis machinery to release Fe atoms [11]. The radioresistant bacterium *Deinococcus radiodurans* induces genes related to iron uptake, cysteine biosynthesis, protein sulfide stress and several DNA repair systems [12]. Finally, exploration of *Pseudomonas brassicacearum* intraclonal adaptation revealed a reorganization of the cell wall to limit Cd import, a production of polyamines to counteract Cd-induced damages and a downregulation of genes involved in motility [13]. To date, among Archaea, only haloarchaeal strategies to withstand stress from transition metals have been investigated [14] and no microarray approach has been performed to analyze heavy metal transcriptomic response of microorganisms belonging to the third domain of life, the Archaea [15], even though several of them are known to live in metal-rich ecosystems.

Metal concentrations in deep-sea hydrothermal chimneys are in the range of 10–40 µM for Cu, 20–2000 nM for Co and 40–3000 µM for Zn [16], [17], [18], and anaerobic and thermophilic microorganisms isolated from a vent at Lau Basin exhibited high resistance to several metals (Cd, Zn, Co and Ni; [19]). The minimal inhibitory concentration (MIC) of cadmium chloride (CdCl_2_) for several *Thermococcus* species is higher than 1 mM [19] but the strategies developed by thermophiles to live in such environments are largely uncharacterized [20]. Moreover, metal resistance is not shared by all organisms thriving in these biotopes since several hyperthermophilic bacteria are found to be highly sensitive to this metal [19]. Therefore, hyperthermophilic archaea have developed strategies to face not only high temperatures but also chemical injuries. Heavy metal tolerance does not only rely on cellular defense processes, but also on the vent fluid’s sulfide-rich content acting as inorganic ligands to complex the metals [21]. These complexes decrease metal bioavailability and hence could partially reduce toxicity, but do not explain metal tolerance discrepancies among the Archaea. Although a growing interest has emerged in recent reports [22], [23], heavy metal resistance mechanisms in *Thermococcales* are largely unknown, in particular for Cd.

In this work, we used as a model *Thermococcus gammatolerans* EJ3^T^, a hyperthermophilic archaeon isolated from a gamma irradiated enriched culture of microorganisms collected in a North Pacific hydrothermal vent [24]. We sequenced and annotated its genome and the proteome content has been analyzed from exponential and stationary phase cells [25]. Here, we investigated the tolerance of *T. gammatolerans* to several metals by measuring the MICs for Cd, Co, Zn, Ni and Cu. The global cellular responses following Cd exposure (100 µM & 1 mM) on *T. gammatolerans* exponentially-growing cells were analyzed using microarrays containing one 50-mer oligonucleotide probe for each of the 2157 annotated protein-coding genes (Figure S1). Among the 114 genes revealed by this strategy, several of them encode transporters, and membrane-bound hydrogenase, oxydoreductase and hydrogenlyase complexes. Finally, using a real-time RT-PCR approach, Cd transcriptional patterns were compared with those induced by other stresses (metals, heat shock, γ-Rays) in *T. gammatolerans*.

## Results and Discussion

### *Thermococcus gammatolerans* Exhibits High Resistance to Cd, Co and Zn

We determined for several metals the lowest concentration (MIC) that completely inhibits *Thermococcus gammatolerans* cell growth at 85°C and compared the data with those from other *Thermococcaceae* such as *Thermococcus litoralis, Thermococcus stetteri* and *Thermococcus celer* [19]. Since a rich-medium culture contains several components able to chelate metals (i.e. glutathione), MIC experiments were performed in artificial seawater (ASW) with limited calcium and phosphate concentrations ([26], [27], see methods) supplemented with amino acids and cystine (ASW-AA-Cyst) or with yeast extract, peptone and cystine (Llanos' medium, [19]). Moreover, sodium sulfide usually used to achieve total anaerobiosis, was replaced by ascorbic acid to reduce metal precipitation [19], [21]. In ASW-AA-Cyst medium, *T. gammatolerans* cell density reaches 2×10^8^ cells/mL and the doubling time is 270 min. Here, cell growth was scored at 24 hrs (as in [19]) and also at 48 hrs to check that cells did not need more time to recover, but no difference was observed at a metal concentration that completely inhibits cell growth (MIC). The Cd MIC in ASW-AA-Cyst medium was found to be at 2 mM, showing that *T. gammatolerans* tolerates high Cd concentrations (Table 1). A pregrowth of *T. gammatolerans* in the presence of 1 mM Cd did not increase Cd resistance (data not shown) in contrast to what was observed for several *Bacillus* strains [19]. Such a constitutive protection mechanism against a stress was already observed for the archaeon *Pyrococcus furiosus* challenged with H_2_O_2_ [28]. The Cd MIC is 3 mM in the culture conditions described by Llanos *et al*. [19] but this medium contains yeast extract and peptone that could chelate metals. This also may explain why MICs observed in Llanos’ medium for other metals (Zn, Ni and Cu) are somewhat higher than in ASW-AA-Cyst medium (Table 1). We also tested *T. gammatolerans* resistance to Co and Zn, two other metals found in hydrothermal environments [16], [17], [18] and showed that whatever the culture media used to perform these experiments, *T. gammatolerans* is rather more tolerant to Co (2 mM) than other *Thermococcale* species described in [19] (Table 1). *T. litoralis* remains the most Zn resistant *Thermococcale* tested (∼5 mM, Table 1). In contrast to Cd, Co and Zn whose MICs are in the millimolar range, *T. gammatolerans* displays micromolar sensitivity to Ni, Cu and arsenate (AsO_4_; Table 1). *T. gammatolerans* growth is inhibited by 500 µM or 750 µM Ni, depending of the culture medium (Table 1), whereas *T. litoralis, T. celer* and *T. stetteri* tolerate millimolar concentrations of Ni [19]. The tolerance of *T. gammatolerans* to other metals such as Cu and AsO_4_ was also determined, and their MICs are less than 1 mM (Table 1). The lower tolerance to Cu was expected since this metal is known as a highly toxic compound that microorganisms tolerate to a limited degree [26], [29].

**Table 1:** Metals MIC (mM) distribution for several *Thermococcale* species.

Metals	*T. gammatolerans* §	*T. gammatolerans* †	*T. litoralis^#^*	*T. stetteri^#^*	*T. celer^#^*
Cd	**2**	3	1	1	1
Co	**2**	2	1	0.8	0.8
Zn	**2**	3	5	0.5	2
Ni	**0.5**	0.75	1.5	5	5
Cu	**0.5**	0.75	nd	nd	nd
AsO_4_	**0.25**	0.25	nd	nd	nd

Values are the means of three independent experiments, with a standard deviation of 10%.

§ MICs values of *T. gammatolerans* in the ASW-AA-Cyst medium (see Materials and Methods).

† MICs values of *T. gammatolerans* in the Llanos’ medium described in [19].

*#* MICs values of other *Thermococcales* previously published in [19].

**Figure 1:**
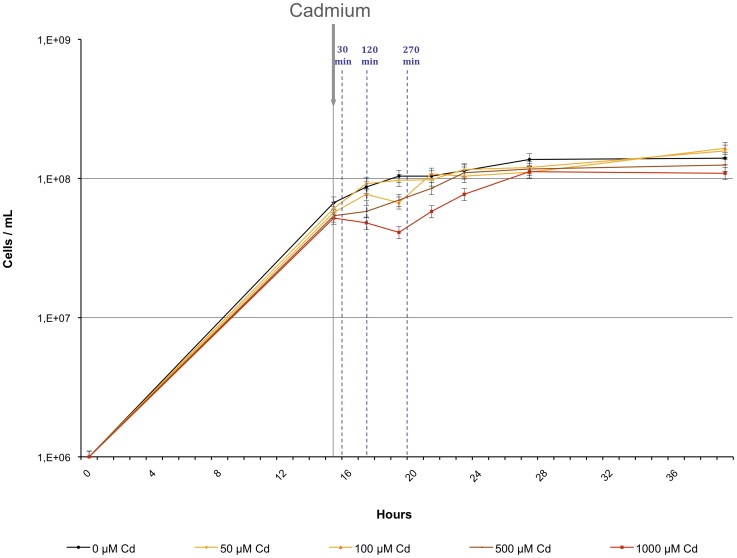
Cd treatment of exponentially growing *T. gammatolerans* cells. Growth of *T. gammatolerans* EJ3^T^ in artificial seawater medium (ASW-AA-Cyst) in the absence of Cd (•), or after addition of 50 µM (♦), 100 µM (▴), 500 µM (-) and 1 mM (▪) Cd. Blue dotted lines indicate the timepoints (30, 120, 270 min) for microarrays analyses. The values are the mean of three independent cultures (standard deviation (SD) ≤10%).

We investigated the impact of a Cd exposure on exponentially growing cells in liquid cultures in ASW-AA-Cyst medium. Whereas the addition of 50 µM or 0.1 mM CdSO_4_ did not modify the growth rate, higher amounts correlated to a concentration-dependent growth decrease compared to an untreated control culture (Figure 1). Exposure to 1 mM Cd triggered a transient growth arrest. Consequently, we determined, by Inductively Coupled Plasma Mass Spectroscopy (ICP-MS, [30], [31]), the amount of Cd that exponentially growing cells retain at 30 min, 120 min, 270 min and 24 hrs under our culture conditions (ASW-AA-Cyst medium) and showed that cells always retain several percents of the amount of Cd (1 mM) added to the culture (Table S1). However, these percentages differ from one culture to another, except for the 24 hrs timepoint. Although the presence of inorganic ligands was minimized in ASW-AA-Cyst culture medium, addition of 1 mM Cd promoted a lot of cell aggregates, containing a few or numerous cells, that probably arise from the reaction of Cd with the sulfur-containing compounds (like H_2_S) produced by cellular metabolism. These aggregates were not disrupted even after three thorough washes in a large volume of fresh medium and only traces of Cd were removed from the cell pellet (Table S1 and Figure S2). In order to eliminate CdS precipitates, the cultures were filtered before centrifugation but the number of single cells or small aggregates remaining in suspension probably diverged in each culture and provided irreproducible data. In spite of these harsh culture conditions, cells were still able to growth (Figure 1), and after 24 hrs they had reached the stationary phase (1.8–2.0×10^8^ cells/ml). The number of cell aggregates dramatically decreased, and the percentage of Cd retained by cells is more reproducible (Table S1). We quantified at 24 hrs the amount of Cd found in each fraction following the protocol described in Figure S2. During growth, Cd is continuously trapped by sulfur components produced by *T. gammatolerans* metabolism and after 24 hrs a large part of the metal (∼ 93%) is found in the CdS precipitate fraction (F4, Figure S2). The remaining amount of free Cd ions in the culture medium is very low (F1; 0.03%). However, the percentage of Cd retained by the cell population (50 mL, 1.8–2×10^8^ cells/mL) is in average 6.7%, thus 67 µM and they remain able to grow in a fresh medium with or without Cd (data not shown). Cd chelation seems not to be performed by metallothioneins or polyphosphate or glutathione, since this archaeon does not encode orthologous enzymes involved in their biosynthesis [2], [5], [6], [25]. Therefore, sulfur probably form the first efficient barrier against Cd toxicity.

### Experimental Design

**Figure 2:**
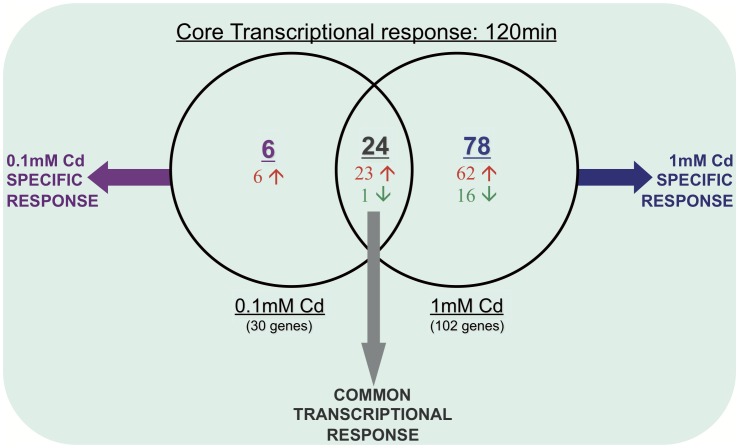
Venn diagram analysis of the transcriptional Cd response after 120min. The diagram shows the numbers of genes regulated (|FC| ≥2 and p-Value ≤ 0.01) during the core transcriptional response (120 min after Cd exposure) for both Cd doses tested (0.1 and 1mM). Upregulated genes: red; downregulated genes: green. For details, see Table S2.

Kinetics of gene expression changes induced by Cd were performed at two different concentrations (0.1 mM and 1 mM) and at 3 timepoints (30, 120 and 270 min). A 0.1mM Cd concentration did not affect the growth rate, whereas 1mM Cd induced a transitory growth arrest for 270 min (Figure 1). Late exponentially growing cells (7×10^7^ cells/mL) were exposed to Cd and were collected after 30 min, 120 min and after 270 min (Figure S1). For each timepoint, four slides containing the 2157 oligonucleotide gene probes printed in duplicate were hybridized (for details see GEO accession number GSE13546 and materials and methods section). The experiment was repeated twice leading to eight data sets per timepoint (four per biological replicate). Microarray analyses monitored 161 transcriptional changes (|Fold Change (FC)|≥2 and p-Value ≤ 0.01, Table 2) in response to Cd exposure corresponding to 114 unique genes i.e. 5.3% of *T. gammatolerans*' gene content (Table 2, Table S2), most of them being upregulated (96/114, 84%; Table S2, Table 2, Figure 2). While about 25% of the upregulated genes exhibited a FC>3 with a maximum of almost 10 for one encoding a conserved hypothetical protein (tg0885, 1mM Cd at 120min), the large majority of the up- and down-regulated genes exhibited a 2 to 3-fold transcriptional change (Table 2) as already described in many archaeal transcriptomic studies [32], [33], [34], [35].

**Table 2:** Overview of Cd transcriptomic data.

[CdSO_4_]	Timepoint (min)	Upregulated	Ratio Max	Downregulated	Ratio Mini	Combined
0.1 mM	30	0 (0)	/	0 (0)	/	0 (0)
0.1 mM	120	29 (5)	3.57	1 (0)	−2	30 (5)
0.1 mM	270	1 (1)	3.11	1 (0)	−2	2 (1)
1 mM	30	21 (5)	4.51	3 (0)	−2.33	24 (5)
1 mM	120	85 (28)	9.56	17 (3)	−3.41	102 (31)
1 mM	270	3 (1)	3.11	0 (0)	/	3 (1)
*Total*	*30, 120, 270*	*139 (40)*		*22 (3)*		*161 (43)*

Number of genes with FC ≥2 or ≤−2 (3- in brackets) after Cd addition (p-Value ≤ 0.01).

After a 30 min Cd treatment, no transcriptional change was monitored at 0.1mM whereas 24 genes responded to 1mM Cd. However, after a 120 min exposure, 30 genes are regulated at 0.1mM Cd and most of them also displayed expression changes at 1 mM (Table 2, Figure 2). These results show that *T. gammatolerans* was sensing Cd not only at a toxic dose (1 mM) but also at a non-toxic concentration (0.1 mM) that does not affect growth. Interestingly, both toxic (1 mM) and non-toxic (0.1 mM) concentrations induce a transcriptomic response predominantly 120min after stress induction (Table 2, Figure 2). Indeed, out of the 114 unique genes identified in this study, 108 are regulated at 120min and 24 genes are common at both doses (Figure 2, Table S2). Among these, 23 are induced, 1 is repressed (tg1897), and almost two-third respond in a dose dependent manner since their respective FC increases in parallel with Cd concentration. Additionally, cell exposure to a toxic dose promotes specific gene regulation (Figure 2, Tables S2). Finally, the number of genes differently expressed 270 min after the beginning of Cd treatment decreases drastically, with 2 and 3 genes being regulated in response to 0.1 mM and 1 mM, respectively (Table 2) suggesting that cells repaired most of the damage and rapidly resumed growth as shown in Figure 1.

**Figure 3:**
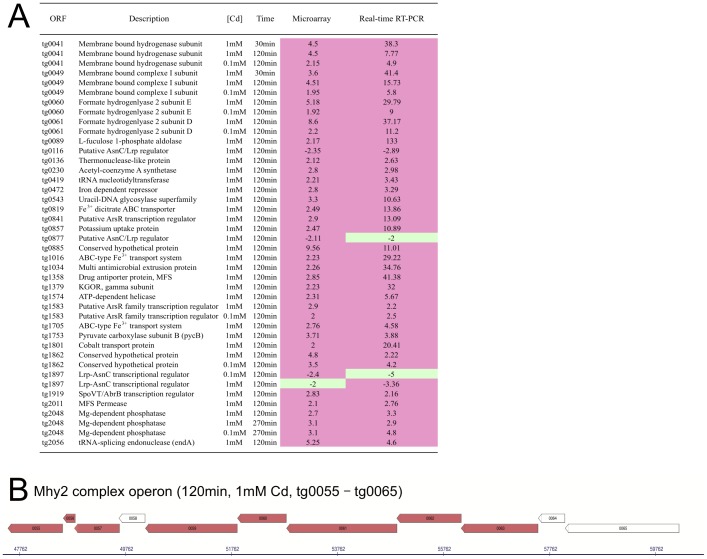
Real time RT-PCR validations and operon regulation during Cd stress. (A) Relative gene expression changes data from microarray analysis and quantitative real-time RT-PCR assays. The relative mRNA amount of 31 genes was measured at different timepoints (30, 120 or 270 min) and two Cd concentrations (0.1 mM or 1 mM). Altogether, 42 real time RT-PCR measurements were determined in triplicate from 2 independent cultures with a SD ≤10%. Red denotes up-regulation (FC ≥2) and green denotes down-regulation (FC ≤ −2). (B) Section of a *T. gammatolerans* chromosomal map illustrating cluster regulations following Cd exposure. The genes operon from tg0055 to tg0065 encoding the formate transporter (Foc) and the formate hydrogenlyase 2 (Mhy2) subunits are coregulated at 120 min after exposure to a dose of 1 mM Cd. Arrows indicate ORFs within the transcript orientation. Upregulated genes: red (FC ≥2 and p-Value ≤ 0.01). Images extracted from http://www-archbac.u-psud.fr/genomes/r_tgamma/tgam_Cd_search.html [25].

Real time RT-PCR was used to validate the transcriptomic data of a set of 31 selected genes encoding hydrogenase subunits, regulators, transporters and conserved hypothetical proteins (Table S3). As illustrated in Figure 3A, their expression changes revealed by real time RT-PCR resulted in values mostly higher than those from microarrays (Table S2), illustrating the broader dynamic range of this method compared to microarray measurements [36], [37], [38]. The biological significance of this genome-wide transcriptional analysis is also strengthened by coregulation of genes belonging to the same operon and encoding protein complexes as, for example genes from tg0055 to tg0065 encoding the formate transporter (Foc) and the formate hydrogenlyase 2 (Mhy2) subunits (Figure 3B, [25]). Finally, 15% of the differentially expressed genes putatively involved in the same biological processes (such as metal homeostasis or drug detoxification), display the same transcriptional regulation tendency although they are dispersed along the genome (Table 3). Thus, real time RT-PCR validations of the microarray data, co-induction of genes belonging to the same operon, and characterization of several differently expressed genes related to metal stresses, support the biological significance of this genome-wide transcriptional analysis.

**Table 3:** List of genes regulated by Cd encoding putative metalloenzymes and transporters.

Gene	Description
tg0342	Membrane transport protein, drug transporter family
tg0472	Fe dependent transcription repressor
tg0560	Predicted permease
tg0819	Fe^3+^ dicitrate ABC transporter, ATP-binding protein
tg0841	Transcriptional regulator, ArsR family related
tg0849	Transcriptional regulator, ArsR family related
tg0857	Potassium uptake protein
tg1016	ABC-type Fe^3+^ transport system, periplasmic component
tg1034	Multi antimicrobial extrusion protein MatE/Na^+^-driven multidrug efflux pump
tg1358	Drug antiporter protein, major facilitator superfamily
tg1583	Transcription regulator, ArsR family related
tg1705	ABC-type transport system, ATPase component, putative Fe^3+^ transporter
tg1801	Co transport protein, cbiQ family
tg1095	Xaa-Pro dipeptidase, metalloprotein
tg2011	Permease, major facilitator superfamily
tg2022	Putative metal-dependent phosphoesterase

In each case (|FC| ≥2; p-Value ≤0.01).

As already observed in previous archaeal transcriptomic studies, about one third of the regulated genes encode proteins of unknown function (Table S2, [32], [33], [34], [35]). Among them, 10 were detected by our large-scale proteomic studies [25]. Several genes could play an important role to counteract the deleterious effects of this heavy metal. Unfortunately, the lack of genetic tools for *T. gammatolerans* does not allow the analysis of the deletion of these genes on cell survival with or without Cd.

### Transporters and Permeases

Three different systems are known to mediate cadmium efflux in archaea and bacteria : P-type ATPases (CadA), resistance-nodulation-cell division (RND)-driven trans-envelope exporters (CzcCBA complex) and cation diffusion facilitators (CDF, CzcD) (for a review, see [39]). Neither homologues of CadA P-type ATPases nor RND CzcCBA complexes are encoded by *T. gammatolerans* genome, and the CzcD orthologue (tg0830) is not affected by Cd exposure in *T. gammatolerans*.

**Figure 4:**
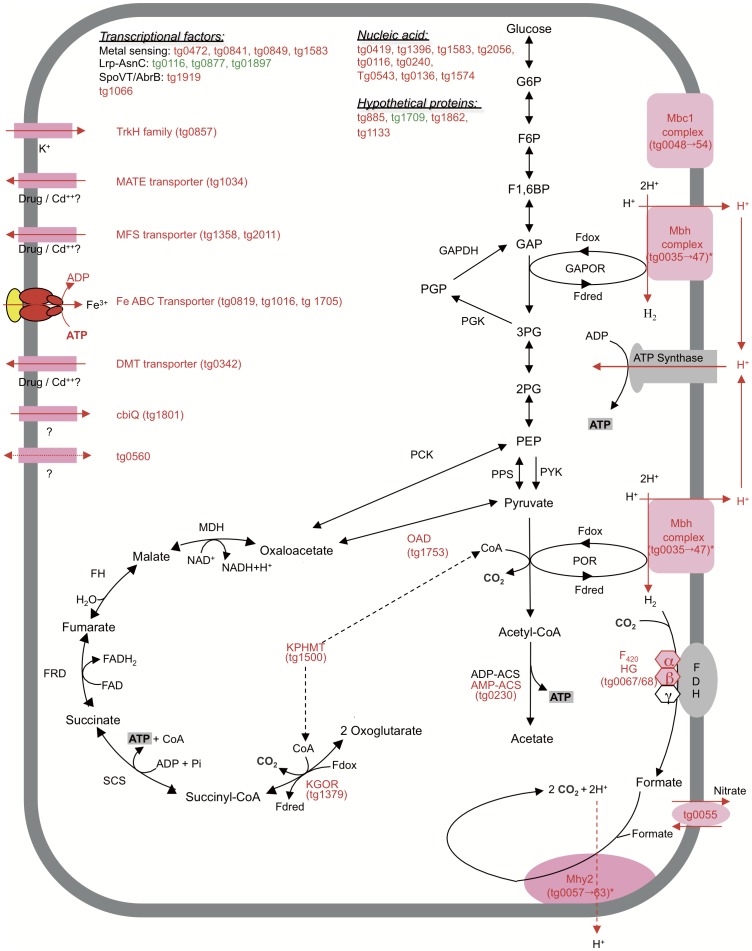
Cd-mediated cellular responses in *T. gammatolerans*. Transcriptional response induced at 120 min when *T. gammatolerans* cells are exposed to 1mM Cd. (ADP-ACS) ADP-forming acetyl-CoA synthetase; (AMP-ACS) AMP-forming acetyl-CoA synthetase; (CoA) coenzyme A; (DMT) drug/metabolite transporter; (F1,6BP) fructose 1,6-bisphosphate; (F_420_ HG) F_420_ reducing dependent hydrogenase; (Fdred) reduced ferredoxin; (Fdox) oxidized ferredoxin; (FDH) formate dehydrogenase; (FH) fumarate hydratase; (FRD) fumarate reductase; (F6P) fructose 6 phosphate; (G6P) glucose 6 phosphate; (GAP) glyceraldehyde 3 phosphate; (GAPDH) glyceraldehyde 3 phosphate deshydrogenase; (GAPOR) glyceraldehyde 3 phosphate ferredoxin oxydoreductase; (KGOR) ketoglutarate:ferredoxin oxydoreductase; (KPHMT) ketopentoate hydroxymethyltransferase; (MATE) multi antimicrobial extrusion protein; (Mbc) membrane-bound complexe; (Mbh) membrane-bound hydrogenase; (MDH) malate deshydrogenase; (MFS) major facilitator superfamily transporter; (Mhy2) formate hydrogenlyase II; (MMM) methylmalonyl-CoA mutase; (OAD) oxaloacetate decarboxylase; (2PG) 2 phosphoglycerate; (3PG) 3 phosphoglycerate; (PCK) phosphoenolpyruvate carboxykinase; (PEP) phosphoenolpyruvate; (PGK) phosphoglycerate kinase; (PGP) 2,3 bisphosphoglycerate; (POR) pyruvate:ferredoxin oxidoreductase; (PPS) phosphoenolpyruvate synthase; (PYC) pyruvate kinase; (SCS) succinyl-CoA synthetase. *tg0041 and tg0061 genes are also induced at 0.1 mM Cd. ORF item is indicated under brackets. Red: upregulated genes (FC ≥2 and p-Value ≤ 0.01); green: downregulated genes (FC ≤ −2 and p-Value ≤ 0.01).

However, the transcriptional response after a 120 min treatment with 1 mM Cd reveals that 11 out of the 82 genes encoding putative transporters and permeases [25] are upregulated (Figure 4, Table S2). The gene tg1358 codes for a drug antiporter member of the major facilitator superfamily (MFS) that uses the proton motive force for drug active efflux in *Mycobacterium smegmatis* [40]. Our analysis also revealed that 1 mM Cd induces expression of tg1034, encoding a MATE protein (Multi Antimicrobial Extrusion family), a Na^+^-driven multidrug efflux pump mediating resistance to a wide range of cationic dyes, aminoglycosides and other drugs [41]. The orthologue of tg1034 in the archaeon *Halobacterium NRC-1* (VNG0727C) also displays an upregulation following Co treatment [14]. Finally, 1 mM Cd also induced the expression of genes coding for permeases related to drug/metabolite transporters (DMT; tg0342 [42]) and to the major facilitator superfamily (MFS; tg2011). The upregulation of tg1801, unique in *T. gammatolerans*, encoding CbiQ a putative cobalt transport protein (Figure 4, Table S2) remains unclear, as the *T. gammatolerans* genome does not code for a whole CbiNMQO transport system [43], [44]. It has been recently shown that CbiO (BioM, tg1802) and CbiQ (BioN, tg1801) together with BioY (tg1804) are subunits of a tripartite biotin transporter found in bacteria and archaea [44]. BioN, BioM and BioY are clustered on the genome of *T. gammatolerans* but only CbiQ/bioN is regulated in the presence of Cd. We have no evidence that BioN and BioM belong to an operon and BioY is located on the opposite strand and is not regulated. However, all these results suggested that these permeases and transporters could help to detoxify the cell or alternatively that a high amount of Cd disrupts the normal functioning of these membrane proteins leading to an imbalance of various ions. This perturbation is also illustrated by the induction of tg0857 belonging to a family of K^+^/H^+^ symporter (K^+^_(out)_+H^+^_(out)_<$>\raster="rg1"<$>K^+^_(in)_+H^+^_(in)_) as well as tg1059 coding an osmotically inducible protein C (OsmC) rapidly upregulated after a 30 min exposure to 1 mM Cd. It has been reported that *T. kodakaraensis* OsmC protein is overexpressed in response to a saline stress but not under oxidative or heat stress [45]. We show here that the gene encoding this protein is also rapidly upregulated in response to such metal stress. Unfortunately, due to the heterogeneity of the cell sizes (ranging between 0.6 and 1.4 µm [24]) and the presence of cell aggregates generated by Cd, it is not possible to estimate if the cell size is affected by the Cd challenge.

Several genes encoding predicted transcriptional regulators [25] are regulated following Cd exposure (10/71 genes, Table S2). Regulation of putative metal-sensing transcriptional regulators (Errs family, IPR001845) encoded by tg0841, tg0849 and tg1583 within the core transcriptional response (120 min) and at both Cd doses also supports metal homeostasis perturbation (Table S2; [46]). Several members of this family bind to DNA via a winged helix-turn-helix (HTH) motif and dissociate from DNA in the presence of metal ions [47]. Induction of such regulator has already been reported when the cyanobacterium *Anabaena* is exposed to Cd [48].

### Iron Homeostasis

**Figure 5:**
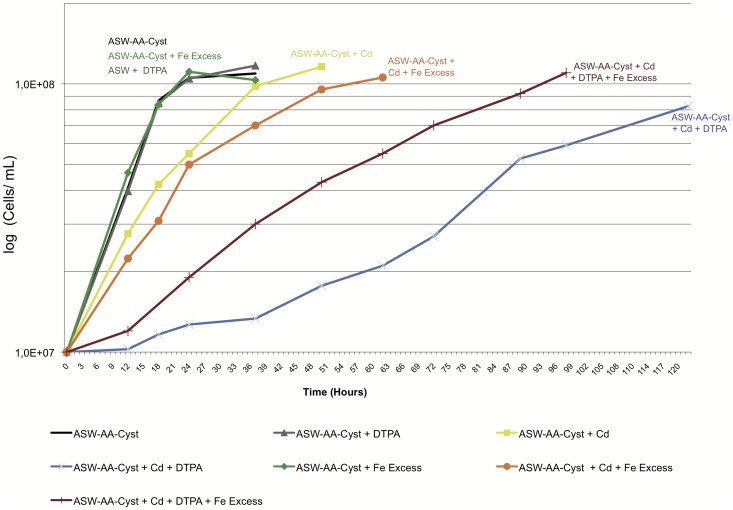
Effect of iron availability on *T. gammatolerans* Cd tolerance. Cd susceptibility was analyzed in ASW-AA-Cyst medium (black line) or in modified ASW-AA-Cyst media supplemented with: 200 µM DTPA (metal chelator, grey line), 100 µM iron (green line), 1 mM Cd (yellow line), 1 mM Cd and 100 µM iron (orange line); 1 mM Cd and 200 µM DTPA (blue line); 1 mM Cd and 200 µM DTPA and 100 µM iron (brown line). The effect of iron availability on Cd tolerance was investigated in each culture medium inoculated with 10^7^ cells/mL and incubated at 85°C on a reciprocal shaker. Cell densities were scored over time using a Thoma counting chamber. The values are the mean of three independent cultures (SD ≤10%).

Our data suggest that Cd induces iron starvation in *T. gammatolerans* and/or a disruption of iron transporters by an unknown mechanism. At a dose of 1 mM Cd, genes encoding subunits of a putative Fe^3+^ ABC transporter such as tg1016 and tg1705 are upregulated. Moreover, tg0819 encoding the ATP-binding protein of the Fe^3+^ dicitrate ABC transporter [49] is also up-regulated as observed in *T. kodakaraensis* in low-iron medium [50] and in *D. radiodurans* cells challenged with Cd [12]. The transcriptional regulator encoded by tk0107 in *T. kodakaraensis* is thought to control the expression of the major iron acquisition effectors [50]. Interestingly, its orthologue in *T. gammatolerans* (tg0472, 77% identity, 94% similarity) is upregulated at both doses of Cd. To assess the relationship between iron availability and cadmium tolerance in *T. gammatolerans*, we analyzed *T. gammatolerans* growth over several generations, when inoculated at 10^7^ cells/mL and challenged with 1mM Cd either under iron starvation or iron excess (Figure 5). The DTPA (diethylenetriamine-pentaacetic acid), a chelator known to be able to trap free metals like iron [51], was added to ASW-AA-Cyst medium to mimic iron starvation. We first determined the maximum DTPA concentration that does not affect either the growth rate or the final cell density of *T. gammatolerans* under our culture conditions. No growth change was monitored in the presence of 200 µM DTPA (Figure 5) whereas higher concentrations (250 µM) led to a reduced growth (data not shown). Therefore, even though DTPA trapped free metals, the remaining small amount of free metals or metal-recycling mechanisms was sufficient for growth. We confirmed that iron was not limiting in ASW-AA-Cyst culture conditions since the growth parameters were not changed in cultures performed in a modified ASW-AA-Cyst medium containing a higher iron concentration (100 µM Fe(NH_4_)citrate instead of 40 µM in ASW-AA-Cyst; Figure 5). *T. gammatolerans* growth is reduced but not impaired in ASW-AA-Cyst medium containing 1 mM Cd since the MIC for Cd is 2 mM (Table 1). Increasing the iron concentration (from 40 µM to 100 µM) in the medium did not rescue cells from Cd toxicity (Figure 5, orange and yellow curves) in contrast to what was observed for *D. radiodurans*. In this bacterium, addition of ferric chloride (100 µM) enhances the growth rate and the final cell density when exponentially growing cells were exposed to low Cd doses (25 or 50 µM; [12]). However, we could not challenge cells with an iron concentration higher than 100 µM since such doses became toxic for *T. gammatolerans*, even without Cd. A slightly reduced growth rate is observed at 100 µM Fe with Cd (Figure 5, orange curve) and higher iron concentrations can enhance Cd toxicity.

The presence of both Cd and DTPA in the culture medium results in a drastic reduction of the growth rate (Figure 5). A lag period of at least 12 hrs is required prior to growth recovery and one cannot exclude that growth restart might be the consequence of DTPA degradation by heat-culture condition, leading to iron release. The growth arrest observed in this condition may be the consequence of DTPA-induced iron starvation since an excess of iron partially restores the growth in the same medium (Figure 5). The DTPA (200 µM) effect is only partially balanced by addition of 100 µM Fe(NH_4_)citrate probably because a large amount of Fe remains trapped by DTPA (200 µM). We added to the culture as controls other transition metals (Zn, Ni, Co), at the same high iron concentration (100 µM), and tested if they are able to rescue cell growth in the presence of DTPA and Cd (Figure S3). Zn and Ni were investigated since they are components of various proteins (*e.g.* Ni-Fe hydrogenases, Zn finger proteins). We also tested cobalt, because of the presence of cobalamin (Vitamin B12) added to our culture medium. The ASW-AA-Cyst culture medium was not supplemented with Ni but contains Zn and Co at a concentration of 3 µM and 3.8 µM respectively. However, as shown in Figure S3, none of these metals was able to rescue growth as iron did. The cell growth restarts as the control (DTPA + Cd, Figure S3). Therefore, the most probable explanation is that iron plays an important role for cell rescue as described in several other organisms [11], [12].

*Synechocystis* cells exposed to cadmium also enhance Fe uptake and degrade their photosynthetic apparatus, to provide extra Fe atoms required to repair damaged [Fe-S] clusters [11]. Iron uptake may also be essential in *T. gammatolerans* to repair damaged iron containing proteins (ferredoxins, hydrogenases, oxydoreductases) required for metabolic pathways, energy production and cofactor recycling.

### Hydrogenase, Oxydoreductase and Formate Hydrogenlyase/Reductant Recycling and ATP Synthesis

At a concentration of 1 mM Cd, a large cluster of genes (tg0035–tg0071) organized in 3 operons, is transiently upregulated at 120 min. Interestingly, several ORFs were also found up-regulated after a 30 min of exposure (Table S2) suggesting a rapid cell response.

First, the tg0035–tg0047 operon encodes the membrane-bound NiFe hydrogenase Mbh found in many *Thermococcales* [25], [52], [53], [54]. This complex oxidizes reduced ferredoxins to maintain metabolic flow and produce H_2_ in the absence of sulfur with the reduction of protons (3H^+^ +2e^−^  =  H_2_+H^+^). The surplus of protons is exported to generate the proton gradient used by the A_0_A_1_-ATPase for ATP synthesis (Figure 4; [52]). Under our experimental assays, *T. gammatolerans* mainly used the amino acids as carbon source and converted them into carboxylic acids [25]. In the presence of sulfur (elemental sulfur or cystine), a second membrane-bound complex Mbx re-oxidized the reduced ferredoxins and is thought to transfer electrons to NAD(P) thus producing NAD(P)H, that is subsequently used by the sulfur reducing enzyme NSR to convert S° into H_2_S [52], [55]. As for Mbh, the surplus of protons is exported to generate the proton gradient used for ATP synthesis (Figure 4). The results presented here show that cells transiently stop to grow (0–270 min) at increasing dose of Cd (Figure 1). One of the presumed mechanisms of Cd toxicity is the replacement of the cognate iron in the membrane-bound Ni/Fe subunits that re-oxidize reduced ferredoxins [3]. The presence of Cd also induced a rapid formation of a CdS precipitate thus reducing the sulfur bioavailability. Therefore, the transient growth arrest should be a consequence of an excess of reductant into the cells and a reduced production of ATP.

In the phylogenetically close *Thermococcale* species *P. furiosus*, transcription of Mbh and Mbx/NSR complexes is antagonistically regulated following S^o^ addition leading to a dramatic repression of Mbh genes and an induction of NSR and Mbx genes [55]. Their respective expression is under the control of the same regulator SurR (PF0095) that acts as an activator of Mbh and as a repressor of Mbx and NSR [56]. However, only the Mbh operon appeared to be upregulated since no antagonistic transcriptional regulation of NSR or Mbx subunits was observed during our transcriptomic kinetic assays. Such discrepancy has already been observed with *P. furiosus* cellobiose-grown cells in which regulation of the expression of Mbh and Mbx is not the on/off mechanism that appears to be present in maltose-grown *P. furiosus* cells [57]. Recently, it has been shown that the oxidation of SurR when the protein is incubated with a colloidal S° solution, dramatically decreases the affinity of the protein for DNA [58]. The homologue of SurR in *T. gammatolerans* is encoded by tg1303 (56% identity, 67% similarity). Indeed, if the transcriptional role of SurR is conserved in *T. gammatolerans*, the transient loss of sulfur could keep SurR under two forms (oxidized and reduced) that would trigger Mbh genes activation to maintain/enhance re-oxidization of reduced ferredoxins, proton translocation and energy biosynthesis.

The second part of the upregulated cluster is composed of the locus (tg0048–tg0054) that codes for a unique membrane-complex among Archaea named Mbc1, composed of proteins that allow Mbh anchorage to the membrane and a MbhH-like subunit whose role in Mbh is unclear (Figure 4, Table S2, [25]). Mbc1 could be a vestigial complex that lost several subunits during evolution. Alternatively, it could interact with other unknown proteins ensuring an adaptation to the environment.

The last part of this cluster (tg0055–tg0065) encodes homologues of formate hydrogenlyase subunits found in *Thermococcus onnurineus* (Mhy2, Figures 3 and 4; [59], [60]). *T. gammatolerans* and *T. onnurineus* are able to grow on formate [60]. In formate-containing medium, *T. onnurineus* expresses these formate hydrogenlyase-encoding genes and the corresponding proteins were abundantly detected relative to growth on starch or CO [60], [61]. The formate hydrogenlyase (tg0055–tg0065) is probably able to catalyze formate oxidation to carbon dioxide (CO_2_) and molecular hydrogen coupled with proton translocation that increases ATP synthesis [60]. We present here the first evidence that formate hydrogenlyase expression is not only regulated by metabolic substrates but also by a wide range of stress including metal stresses, heat shock and γ-Rays (Table 4).

**Table 4:** Quantitative real-time RT-PCR expression data of 27 *T. gammatolerans* genes following various stresses (Cd, Zn, Ni, heat shock and γ-Rays).

ORF	Description	Cd *(1mM)*	Zn *(1mM)*	Ni *(250µM)*	Heath Shock *(95°C)*	γ-Rays *(2500Gy)*
*Transport*						
tg0857	Potassium uptake protein, TrkH family	**10.89**	**7.64**	1.24	**−4.39**	**−**1.11
tg1034	Multi antimicrobial extrusion protein	**34.76**	**19.27**	**7.49**	**−2.34**	1.16
tg1358	Drug antiporter protein, MFS	**41.38**	**32.05**	**9.10**	**−**1.50	1.11
tg1801	Cobalt transport protein, cbiQ family	**20.41**	**27.52**	**5.07**	**−**1.37	**−**1.09
tg2011	MFS Permease	**2.76**	**4.37**	**2.13**	1.08	**2.31**
*Iron-related genes*						
tg0472	Iron dependent repressor	**3.29**	1.22	1.40	**2.18**	**−2.42**
tg0819	Fe^3^ dicitrate ABC transporter	**13.86**	**29.82**	**9.51**	**−**1.04	1.33
tg1016	ABC-type Fe^3^ transport system	**29.22**	**10.75**	**12.51**	**−2.79**	1.19
tg1705	ABC-type Fe^3^ transport system	**4.58**	**12.14**	**3.92**	**−**1.37	1.27
*Hydrogenases/membrane-bound complexes*						
tg0041	Membrane bound hydrogenase subunit	**7.77**	**4.08**	**15.10**	**−26.40**	1.30
tg0049	Membrane bound complex subunit	**15.73**	**20.44**	**5.07**	**−5.40**	**−4.01**
tg0060	Format hydrogenlyase 2 subunit	**29.79**	**36.77**	**11.21**	**−12.30**	**−2.50**
tg0061	Formate hydrogenlyase 2 subunit	**37.17**	**32.98**	**20.46**	**−15.48**	**−2.45**
*Transcriptional factors*						
tg0116	Putative AsnC/Lrp regulator	**−2.89**	**−11.79**	**−**1.85	**−6.17**	**−**1.40
tg0841	Putative ArsR transcription regulator	**13.09**	**2.99**	**39.40**	1.30	**2.70**
tg0877	Putative AsnC/Lrp regulator	**−2.00**	**−13.69**	1.04	**−**1.73	**−2.90**
tg1897	Lrp-AsnC transcriptional regulator	**−3.36**	**−18.83**	**−2.05**	1.23	**−**1.51
tg1919	SpoVT/AbrB transcription regulator	**2.16**	1.02	**5.72**	**−**1.59	**−**1.64
*Central metabolism*						
tg0230	Acetyl-coenzyme A synthetase	**2.98**	**18.83**	0.96	**2.80**	**5.03**
tg1753	Pyruvate carboxylase subunit B	**3.88**	**10.91**	1.17	**−2.63**	**−3.36**
*Nucleic acid repair*						
tg0136	Thermonuclease-like protein	**2.63**	**−**1.91	**4.64**	**−5.05**	**−**1.53
tg0419	tRNA nucleotidyltransferase	**3.43**	**17.21**	**−**1.18	**−**1.63	**2.58**
tg0543	Uracil-DNA glycosylase superfamily	**10.67**	**8.49**	**2.68**	1.01	**−**1.99
tg1574	ATP-dependent helicase	**5.67**	**16.03**	1.11	**−**1.21	**2.17**
tg2056	tRNA-splicing endonuclease	**4.60**	**3.36**	**−**1.91	**3.05**	**−**1.34
*Unknown*						
tg0885	Conserved hypothetical protein	**11.01**	**4.70**	**3.73**	**−**1.47	**−**1.25
tg1862	Conserved hypothetical protein	**2.22**	**4.21**	**−**0.79	1.33	**−**1.53

Bold numbers denote regulated genes |FC|≥2.

Values are the means of two independent biological samples each performed with two technical replicates (SD of 10%).

In addition, our data also suggest that the archaeon induced some specific metabolic steps that produce ATP (Figure 4, Table S2). The induction of the oxaloacetate decarboxylase (OAD; tg1753) encoding gene leads to pyruvate production. Pyruvate could be converted by the pyruvate oxydoreductase (POR). The production of acetyl-CoA required coenzyme A whose biosynthesis pathway is also active, as suggested by the induction of tg1500 encoding a ketopentoate hydroxymethyltransferase (KPHMT) corresponding to the first step of this pathway [62]. Subsequently, acetyl-CoA is transformed by ADP-forming acetyl-CoA synthetase (ADP-ACS) to produce acetate and ATP [63]. Tg0230, that currently only shares orthologues among other *Thermococcales* with *Thermococcus sp* AM4, and which encodes an AMP-forming acetyl-CoA synthetase (AMP-ACS; acetyl-CoA + AMP + PPi ↔ acetate + ATP + CoA), is also upregulated, likely leading to an increased ATP production (Figure 4). Interestingly, even at a low dose of Cd (0.1 mM), either tg1500 (FC:+1.89, p-Val: 2e-05) and tg0230 (FC:+1.93, p-Val: 2e-05) should also respond to a slight stress. The tg0230 gene encodes one of the most abundant proteins in *T. gammatolerans* [25]. To investigate if tg0230 transcriptional induction is correlated with acetate accumulation, we quantified acetate levels (Acetate Kit, Biosentec, France) in supernatants from exponentially growing cells exposed to Cd (1mM, timepoint 120 min) or not. These enzymatic assays showed a slight but reproducible 13% increase of acetate concentration in the supernatant of cells treated with Cd compared to untreated cells supernatant corroborating an increase of ATP production by induction of the AMP-forming acetyl-CoA synthetase.

Altogether, these results suggested that *T. gammatolerans* cells during Cd stress activate several compensatory mechanisms to maintain or enhance proton translocation and energy biosynthesis to repair damage produced by this metal.

### DNA/RNA Repair and Redox Stress

Several genes related to post-transcriptional RNA modification displayed a strong upregulation at both Cd doses (Table S2, Figure 3) such as genes encoding a tRNA nucleotidyltransferase (CAA adding enzyme, tg0419), a SAM-dependent tRNA/rRNA cytosine-C5 methylase (tg1396), and a tRNA-splicing endonuclease endA (tg2056; Table S2). Their upregulations respond in a dose dependant manner since the FCs rise according to increased Cd concentration. These results support the notion that intracellular Cd generates RNA injuries. Intracellular Cd is also known to induce single-strand breakage in DNA [64] as well as to produce genotoxic and mutagenic events by inhibiting various DNA repair processes, especially base excision repair (BER) and mismatch repair (MMR) [65]. The presence of a MMR mechanism in *Thermococcales* is still an open question since no homologue of MutS and MutL are encoded by these organisms [66]. While *T. gammatolerans* is one of the most radioresistant archaea isolated so far and codes for efficient pathways able to rapidly repair huge DNA damages [25], [67], only a few repair genes were found upregulated (tg0136, tg1574 and tg0543) in contrast to what was found in the radioresistant bacterium *D. radiodurans* challenged with Cd [12]. tg0136 and tg1574 encode a thermonuclease homologue and an ATP-dependent helicase respectively. Finally, tg0543 (upregulated at both Cd doses) encodes an Uracil-DNA glycosylase that excises uracil residues from DNA by cleaving the N-glycosylic bond, initiating the base excision repair (BER) pathway [68].

Several evidences showed that an oxidative stress occurred in various cells exposed to Cd [11], [12]. Cd toxicity has partly been attributed to its ability to induce formation of reactive oxygen species (ROS) [3], [69]. Although *T. gammatolerans*, as other *Thermococcales* species, is well equipped to face oxidative stress [25], none of the related genes appeared differentially expressed. This whole-genome study clearly shows that the transcriptional responses to counteract DNA damage and oxidative stress differ from that of bacteria. These discrepancies may be directly related to their respective features. In contrast to *D. radiodurans*, *T. gammatolerans* is a hyperthermophilic organism growing optimally at 85°C. Maintenance of genome stability and the presence of a pool of active proteins are critical for the survival of organisms especially to those living at high temperature. Most of the DNA repair proteins and protective systems against redox are expressed in *T. gammatolerans* exponentially growing cells and stationary phase cells without additional stress [25]. Moreover, only few DNA repair genes are upregulated when *P. furiosus* cells are irradiated at a γ-Rays dose of 2.5 KGy [32], [33], [34], [35] and the genes involved in redox homeostasis appeared to be constitutively expressed after irradiation and also when *P. furiosus* cells are challenged with H_2_O_2_ [28].

Therefore, under non-saturating damage conditions, the absence of some specific targets involved in the MMR and the constitutive expression of numerous repair and protective systems should be a advantage to withstand the noxious effects produced by Cd in proteins and DNA.

### Comparison of Cd Transcriptional Response with Other Stresses

We monitored by real-time RT-PCR the expression of a set of 27 Cd responsive genes belonging to the functional categories discussed above, following exposure to Cd and other kind of stress (Zn, Ni, heat shock, γ-Rays, Table 4). For further metal stresses, exponentially growing cells were exposed during 120 min to sublethal doses (1 mM Zn or 0.25 mM Ni, MIC 50%). For heat stress, cultures were shocked by shifting incubation temperature from 85°C to 95°C during 15 min, allowing a very strong upregulation of tg1688 (FC ∼ 450; data not shown) encoding the small heat shock protein as observed in *P. furiosus* [35]. For γ-irradiation, exponentially growing cells were exposed to 2500Gy and incubated post-irradiation in a fresh medium at 85°C during 120 min (see methods). At a dose of 2500Gy, 100% survival is observed [67].

Interestingly, different metal stress induced similar expression patterns although the effects of each metal is more or less pronounced but these patterns clearly differ from that of heat shock and γ-irradiation (Table 4). Only three genes (tg0230, tg0841, tg2011) induced by a metal stress also display an upregulation after a heat shock or a γ-irradiation. Zn exposure induced a similar pattern compared to Cd whereas Ni stress regulates only 17 out of 27 genes. This mimetic effect has already been reported in *Synechocystis* [11], since Cd and Zn induce toxicity by exchange with cognate metal in metalloproteins. The permeases and transporters (tg0857, tg1034, tg1358, tg1801, tg2011) were also upregulated in the presence of Zn or Ni, likely highlighting a cell response to other ions imbalance. The overexpression of the predicted metal-sensing transcriptional regulator tg0841 in response to metal treatment, particularly to Ni, supports this hypothesis. This gene does not encode the orthologue of the *Thermococcales* NikR regulator (tg0232) that governs Ni import [70]. Zn and Ni overloads may also promote an iron imbalance, since the three specific iron transporters encoding genes (tg0819, tg1016, tg1705) display a strong upregulation. Interestingly, Cd but not Zn or Ni induced the transcriptional regulator tg0472, orthologous to tk0107 in *T. kodakaraensis*, thought to be involved in the expression of the major iron acquisition effectors [50]. However it has not been shown that the protein is able to bind to the promoters of the genes encoding these effectors. Further analysis will decipher the role of this transcription factor in *Thermococcales* species.

The most upregulated gene (tg1919) at 0.1 mM Cd was also upregulated at 1 mM Cd as well as with 250 µM Ni as shown by qRT-PCR (Table 4 & Table S2). Tg1919 encodes a transcriptional regulator unique among *Thermococcales* and closely related to the SpoVT/AbrB family. In *Bacillus subtilis*, AbrB proteins are transition state regulators that play an essential role in the adaptive capacity and for survival when environmental conditions are deleterious [71]. Moreover, during the core transcriptional response (120 min), several Lrp-like transcriptional regulators (DM1, DM2, DM3 encoded by tg0116, tg1949, tg0877 respectively) belonging to a Feast/famine Regulatory Proteins (FFRPs), were downregulated (Table 4). DM1, DM2 and DM3 regulators from *Pyrococcus sp*OT3 bind specific amino acids [72] to regulate amino acid catabolism [73]. Their targets on the genome remain to be identified as for most of their orthologues in *Pyrococcus sp* OT3 [72], [73], [74]. Our large-scale proteomic analysis showed that most of them were expressed when cells grew in a rich proteinous medium containing S° [25], and we showed here that, among the dozen of *T. gammatolerans* FFRPs regulators, the 3 halves FFRPs displayed the same behavior under metal stress condition. Their repression could be associated with a fine-tuning regulation of several metabolic pathways observed here.

While Cd is known to inhibit base excision repair [66], only tg0543 encoding a DNA glycosidase is specifically regulated by all metal stresses. This gene displays a stronger upregulation under a Cd stress than with an excess of Ni or Zn suggesting that Cd could target the protein, and cell upregulates the gene to prevent DNA damage and subsequent mutagenesis.

Both heat shock and γ-Rays damage cellular components. However, *T. gammatolerans* regulates less than 50% of these 27 genes (11 out of 27 for γ-rays) and most of them display a transcriptional downregulation. During the heat shock, *T. gammatolerans* cells induced tg1688 and downregulated tg1034 that encode a small heat shock protein and a multiantimicrobial extrusion protein respectively as observed in *P. furiosus* (PF1883 and PF1850) after a 1 hr heat shock treatment at 105°C [35]. While metal stresses induce genes encoding Mbh, Mbc1, Mhy2 subunits (tg0041, tg0049, tg0060 and tg0061) likely to compensate replacement of the cognate iron in the membrane bound Ni/Fe subunits used to re-oxidize the reduced ferredoxins [3], heat shock stress and to a lesser extend γ-irradiation exposure lead to a downregulation of these complexes. In these two stresses, *T. gammatolerans* is grown in sulfur containing medium and cells probably re-oxidize the reduced ferredoxins mainly using the Mbx complexes. Therefore, *T. gammatolerans* is likely able to develop a set of specific strategies to re-oxidized cofactors and produce energy according to the nature of the stress and its impact on metabolic pathways.

Altogether, these results reveal that Cd transcriptional pattern display close similarities with other metal expression patterns but the Cd transcriptional response is not a general stress response since only few Cd responsive genes were similarly regulated by heat shock or γ-Rays.

### Conclusion

Deep-sea hydrothermal vents result from seawater infiltration generated by cracks that appear at places weakened by subjacent tectonic activity. *Thermococcales* species thriving in these biotopes are continuously exposed to loads of reduced metals and develop strategies to face such harsh environments. Therefore, *T. gammatolerans* exhibits elevated resistance to several metals as Cd, Co and Zn, and at a lesser extent to others as Ni, Cu and AsO_4_. For the first time in Archaea, we performed a time-course transcriptomic analysis to draw a global response to Cd toxicity in growth conditions close to its natural habitat (artificial seawater, amino acids and cystine as donor of sulfur). Several bacterial and eukaryal transcriptomes showed that sulfur compounds play a key role to trap Cd for detoxifying the cells [7], [8], [9], [12]. The main metabolic pathways of *T. gammatolerans* is based on amino acid catabolism in the presence of sulfur that continuously produces these compounds and may establish the first efficient barrier of protection against metals [21]. Our strategy highlighted that *T. gammatolerans* reprograms, at a sublethal Cd dose, a hundred genes which are predominantly up-regulated 120 min after exposure. Previous archaeal transcriptional analyses also highlighted a regulation of only 50–150 genes following other stresses (cold shock, heat shock or H_2_O_2_ exposure; [28], [33], [35]). One possible explanation is that several protective mechanisms known to be inducible in mesophiles appear constitutively expressed in hyperthermophilic archaea, some of them ensuring a natural protection against this metal. Cd exposure often induces an oxidative stress, which results in damage of proteins and DNA. The high expression level in *T. gammatolerans* of accurate repair mechanisms as well as systems involved in oxygen detoxification and redox homeostasis provides efficient defenses against Cd. The induction of several permeases and transporters helps to detoxify cells and counteracts metal homeostasis perturbations. This work also shows that *T. gammatolerans* cells develop strategies already observed with other organisms as Fe uptake (*Synechocystis*, *D. radiodurans* [11], [12]) and formate transporter induction (*P. brassicacearum* [13]) but also new archaeal specific pathways as the induction of membrane bound Mbh, Mbc1, Mhy2 complexes. Most of these pathways are also activated under metal stresses promoted by Zn or Ni and likely characterize common ways to face various metal stresses to which *T. gammatolerans* is naturally exposed.

## Materials and Methods

### Strain, Media, Growth

*T. gammatolerans* EJ3^T^ was kindly provided by D. Prieur (Université de Bretagne Occidentale, Institut Universitaire Européen de la Mer, 29280 Plouzané, France). Strain EJ3^T^ was grown in serum bottles, 1L Schott bottle or in Hungate tubes, under strictly anaerobic conditions at 85°C in artificial seawater medium containing amino acids, vitamins and cystine (ASW-AA-Cyst) or in complex organic medium with sulfur (VSM- S°).

The artificial seawater medium (ASW-AA-Cyst) contains reduced calcium and phosphate amount and cystine instead of inorganic sulfur (S°) as electron acceptor to minimized metal complexation [19]. The ASW medium contained, per liter of distilled water: NaCl 20g; MgCl_2_.6H_2_O 3g; MgSO_4_.7H_2_O 6g; (NH_4_)_2_ SO_4_ 1g; NaHCO_3_ 0.2g; CaCl_2_.2H_2_O 0.3g; KCl 0.5g; KH_2_PO_4_ 0.42g; NaBr 0.05g; SrCl_2_.6H_2_O 0.02g; Fe(NH_4_)citrate 0.01g; Pipes 3g. This basal ASW medium was supplemented with 0.5 mL per liter of modified 10X Wolfe's trace minerals stock solution (nitrilotriacetic acid 15g, CoCl_2_ 1g; ZnSO_4_ 1g; CuSO_4_.5H_2_O 0.1g; AlK(SO_4_)_2_ 0.1g; H_3_BO_3_ 0.1g and NaMoO_4_.2H_2_O 0.1g per liter; [75]), vitamin mixture (5 ml/L, [75]), 20 amino acids (alanine 75 mg; arginine 125 mg; asparagine 100 mg; aspartic acid 50 mg; glutamic acid 200 mg; cysteine 250 mg; glutamine 50 mg; glycine 200 mg; histidine 100 mg; isoleucine 100 mg; leucine 100 mg; lysine 100 mg; methionine 75 mg; phenylalanine 75 mg; proline 125 mg; serine 75 mg; threonine 100 mg; tryptophan 75 mg; tyrosine 100 mg and valine 50 mg per liter), cystine (2 g/L) and ascorbic acid (1 g/L) to reduce the oxygen dissolved in the medium.

The complex organic medium (VSM- S°) is composed of 20g/l NaCl, 0.25g/l KCl, 0.05g/l NaBr, 0.02g/l boric acid, 0.01g/l SrCl_2_.6H_2_O, 0.5g/l trisodium citrate, 3g/l Pipes, 1g/l yeast extract, 4g/l bactotryptone, 5 ml MgSO_4_ 20%, 1 mL CaCl_2_ 5%, 1 mL KH_2_PO_4_ 5%, 2g/L inorganic sulfur S° (sulfur flowers, Fischer Scientific). Na_2_S at a 0.1% final concentration was added to reduce the oxygen dissolved in the medium.

For both ASW-AA-Cyst and VSM- S° media, NaOH was added to adjust the pH to 6.9 before sterilization. Air contained in the bottles or tubes was first removed using a vacuum and replaced by N_2_ (100%; Air Liquide, France). Absence of O_2_ was followed through disappearance of resazurin sodium salt (1 mg/L) pink color included in the media. Cell culture densities were measured by optical microscopy (Olympus BH-29) using a Thoma counting chamber (Microgravure Precis).

### Metal Susceptibility Testing and Metal Stress

CdSO_4_, CoSO_4_, ZnSO_4_, NiSO_4_, CuSO_4_, ArsSO_4_ and DTPA (diethylenetriamine-pentaacetic acid) were purchased from Sigma. Stock solutions were prepared in MilliQ water, filter sterilized and stored in the dark.

The minimum inhibitory concentration (MIC) determinations were performed in ASW-AA-Cyst medium as described above to avoid metal complexation and also in Llannos’ medium (containing yeast extract: 1 g/L, peptone: 4 g/L as carbon sources and not amino acids as in our medium, the detailed composition is provided in [19]). Fresh exponential culture was used to inoculate Hungate tubes containing 3 mL of a given medium at a cellular density of 5×10^5^ cells/mL with different metal concentrations (100, 250, 500, 750, 1000, 1500, 2000 and 3000µM). To prevent metal precipitation with hydrogen sulfide produced by *T. gammatolerans* preculture, cells were harvested and washed with sterile basal medium before inoculation. The MIC was defined as the lowest concentration of metal that inhibits growth after incubation at 85°C on a reciprocal shaker. Cell growth was scored at 24 hrs (as in [19]) and also at 48 hrs to check that cells did not need more time to recover. Growth medium without metal and inoculated with *T. gammatolerans* was used as a positive growth control. Growth was measured by direct counts on a Thoma counting chamber. Experiments were repeated in triplicate.

For Cd microarray analyses (Figure S1), large volumes of exponentially growing cells (600 mL in 1L Schott bottles) were treated with Cd (0.1 mM or 1 mM) before sampling 30, 120 and 270 min after Cd addition. Cells were filtered to eliminate cystine (MicraCloth Calbiochem, La Jolla, CA, USA) and pelleted by centrifugation (4000 g, 20 min at 4°C), immediately frozen and stored at −80°C until further processing. Each culture was repeated twice.

For Real Time RT-PCR analyses, cells challenged by Zn or Ni were obtained using large volumes of exponentially growing cells (600mL in 1L Schott bottles) treated with Zn (1mM, MIC 50%) or Ni (0.25mM, MIC 50%) before sampling 120 min after metal addition. Cells were filtered to eliminate cystine (MicraCloth Calbiochem, La Jolla, CA, USA) and pelleted by centrifugation (4000g, 20min at 4°C), immediately frozen and stored at −80°C until further processing. Each culture was repeated twice.

### Heat Shock and Gamma Irradiation Experiments

For the heat shock experiment, a dozen of 50 mL VSM-S° medium serum bottles were inoculated at 5×10^5^ cells/mL and incubated at 85°C. When cell density reached ∼5×10^7^ cells/mL, 6 serum bottles cultures were rapidly heated at 95°C (water bath) and incubated during 15 min while the other serum bottles were kept at 85°C. Cultures (95°C and 85°C) were harvested by centrifugation, immediately frozen and stored at −80°C until needed.

For γ-Rays irradiation assays, exponentially growing cells were rapidly cooled at 4°C, filtered to remove sulfur compounds from the VSM-S° medium and concentrated 10 times by centrifugation [67]. Briefly, cells were exposed to a dose of 2500Gy (100% survival, [67]) using a ^137^Cs γ-Rays source (40 Gy/min, IBL637 CisBio International, Institut Curie, Orsay, France). The same number of non-irradiated cells was kept in ice during irradiation. Irradiated and non-irradiated control cells were then incubated 120 min at 85°C in fresh VSM-S° medium on reciprocal shaker (as described in [67]) before centrifugation.

### RNA Extraction and cDNA Synthesis

For microarray experiments, total RNAs were extracted with TriReagent™ solution (Sigma) and contaminating DNA was digested using DNA-*free*™ DNase (Ambion) following the manufacturer's instructions. RNA quantity and quality were assessed using a Nanodrop BD-1000 spectrophotometer (Nanodrop Technologies) and Experion Automated Electrophoresis System (Bio-Rad Laboratories), respectively. The reverse transcription reactions were performed with 400 units of Superscript II reverse transcriptase (Invitrogen), RNA (20 µg), random hexamers (6 µg), 1.5 mM dATP, dCTP, and dGTP, 1.2 mM dTTP, and 0.3 mM amino allyl-dUTP (Sigma) for 1.5 hrs at 42°C. RNA was then hydrolyzed during 15 min at 37°C by 2 units of RNase H. The cDNAs were purified with Microcon-G30 (Millipore), dried and resuspended in 10 µL water. Indirect labeling of cDNA (amino allyl-dUTP) with either cyanine3 (Cy3) or cyanine5 (Cy5) monofunctional NHS-ester (Amersham Bioscience) occurred after the reverse transcription. cDNAs, mono reactive Cy3 or Cy5 dye and 1µl of 0.5M pH9 sodium bicarbonate are mixed together and incubated 1 hr at room temperature in the dark for the coupling reaction. The fluorescently labeled cDNAs were purified using a Nucleospin Extract II kit (Macherey-Nagel) and dye incorporation was measured using Nanodrop BD-1000 spectrophotometer (Nanodrop Technologies). The two labeled cDNAs were combined, mixed with 10 µg polyd(A) and 10 µg yeast tRNA, precipitated with ammonium acetate/ethanol and resuspended in 50 µL of hybridization buffer (2X SSC, 0.1% SDS, 0.1% salmon sperm DNA).

### Microarray Hybridization, Scanning and Data Analysis

After a denaturation step for 2 min at 95°C, the concentrated Cy3 and Cy5 cDNA were dropped onto glass slide microarrays and incubated in a hybridization chamber (Corning) for 17 h at 50°C. Four post-hybridization washes of 5 min were carried out at room temperature by incubating slides under agitation in a 2× SSC/0.1% SDS solution, in a 1× SSC solution, in a 0.2× SSC solution and in a 0.05× SSC solution, respectively. The slides were dried by centrifugation. Fluorescent signals were detected by a GenePix™ 4000B laser microarray scanner (Axon Instruments) with a 10 µm resolution. All slides were scanned using 100% laser power and PMT voltage auto-adjustment. The resulting 16-bit images were analyzed using GenePix Pro 6.0 software (Axon Instruments). Spots or areas of the array with obvious blemishes, deformation or dust were manually flagged and excluded from subsequent analysis. Raw data overview and preprocessing were processed using MANGO (MicroArray Normalization tool of GodMap) software [76], an R script that allows the user to make a complete differential analysis of several two-color microarrays from raw data files. A non-linear LOWESS normalization [77] was applied to the raw data. Each gene expression ratio is reported as an average value, and statistical significance was calculated using the Student’s t-test. A 2-fold change expression cut-off for the ratio experiments was applied together with a p-Value<0.01 rendering the microarray analysis highly significant and reproducible.

All the results are available via an integrated database (http://www-archbac.u-psud.fr/genomes/r_tgamma/tgam_Cd_search.html) with visualization tools developed for this project [25]. The microarray data described herein have been deposited in the MIAME compliant Gene Expression Omnibus database (GEO, http://www.ncbi.nlm.nih.gov/geo/) under the accession number GSE13546 [78].

### Array Design and Manufacturing

One 50-mer oligonucleotide probe for each of the 2157 annotated protein-coding genes was designed with Primer3 Software [79] using default parameters except for probe length, which was set between 49 and 51 nucleotides, and primer melting temperature set between 70 and 90°C. Oligonucleotides cross match to other genome regions was checked with blastn. Oligonucleotides defined by Primer3 were synthesized by Sigma-Proligo. The microarray were manufactured at CEA, Evry (France) with the MicroGrid microarrayer from Biorobotics, under strictly controlled temperature and humidity. Oligonucleotide probes were printed onto commercial HydroGel slides (Schott, Germany) under quality controlled conditions, as previously described [80], within the “Toxicologie Nucléaire Environnementale” research program (www.toxnuc-e.org).

### Expression Profiling by Real-time RT-PCR

Total RNA was extracted from *T. gammatolerans* as described above. Reverse transcriptions were performed with 5 µg of total RNA using reverse PCR primer as gene specific primer (Table S3) and SuperScriptII™ as reverse transcriptase (Invitrogen, Carlsbad, CA). PCR primer pairs were designed using Primer3 software [79] with standard parameters and calibrated with genomic DNA. cDNA templates were diluted to 1/500 for expression measurements. Standard conditions were used with LightCycler^®^ FastStart DNA Master^PLUS^ SYBR green I (Roche, DNA denaturation at 95°C for 10 min followed by 40 cycles of 95°C for 10s, 60°C for 10s and 72°C for 15s) using the LightCycler System (Roche Diagnostics). The specificity of each PCR reaction was checked by measuring fluorescent signals during melting curve analysis (PCR product heated from 65°C to 95°C continuously and slowly, 0.1°C s^−1^). Gene expression was calculated relative to the transcripts levels of the gene rpoB (tg1926, encoding RNA polymeraseβ subunit) using the formula 2^−ΔΔCt^ [81]. RpoB is constitutively expressed in our experimental conditions according to microarray data.

### Acetate Measurements

*T. gammatolerans* culture supernatants (1 mL) were deproteinized using 100 µL of ice cold 35% perchloric acid leading to protein precipitation after centrifugation (6000g for 2 min). The supernatants were transferred into a new tube and neutralized with 100µL of ice cold 7N KOH. The sample are then centrifugated at 6000g for 10min, the supernatants are filtered (Mini-Uniprix PVDF filter, Whatmann) and directly used. Acetate assays were performed according to the manufacturer's instructions with an enzymatic kit based on the monitoring of NADH production at 340nm using acetyl-CoA synthetase, citrate synthase, and L-malate dehydrogenase (Biosentec, Toulouse, France).

### Cd Analysis by ICP-Q MS

Late exponentially growing cells (3 independent cultures in 50 mL ASW-AA-Cyst medium) were challenged with 1 mM Cd during 30 min, 120 min, 270 min or 24 hrs. Then, cells were filtered and pelleted by centifigation (4000g, 20 min at 4°C). The pellet was thoroughly resuspended three times in 15 mL of fresh ASW-AA medium to eliminate free Cd ions. After centrifugation (4000g, 20 min at 4°C), the three washing supernatants were pooled for analysis. To quantify Cd retained by *T. gammatolerans*, cells were resuspended in 3 mL ultra pure HNO_3_ (69%) purchased from Sigma-Aldrich (St. Quentin Fallavier, France), and were sonicated in a Branson 1210 bath during 1 hr. The solution was diluted 1000 times in HNO_3_ 2% prior analysis by ICP-MS. The washing supernatants were diluted 10 times in HNO_3_ 2% prior analysis by ICP-MS. Rhodium was used as internal standard and quantifications were performed by external calibration [30], [31] (Agilent 7500 ICP-MS, Agilent Technologies, Tokyo, Japan).

## Supporting Information

Figure S1 **Experimental design of microarray approach.** *T. gammatolerans* exponentially growing cultures were exposed to 0.1 or 1 mM Cd. As a control, cultures without Cd (w/o Cd) were grown in parallel. Time course aliquots were collected 30, 120 and 270 min after Cd exposure. RNA was purified and reverse transcribed. Labeled cDNA were hybridized onto microarrays containing oligonucleotide probes for all ORFs printed in duplicate (GEO accession number GSE13546). The Cd transcriptional response was monitored in two independent cultures for each condition and each biological replication was hybridized twice on a microarray in a dye swap (ds) manner leading to a total of 8 data points per condition and per gene.
(TIFF)

Figure S2 **Quantification by ICP-MS of Cd.** *T. gammatolerans* exponentially growing cells (50 mL) were challenged at a cell density of 5×10^7^ cells/mL with 1 mM Cd during 24 hrs. Following Cd treatment, cells were filtered and pelleted by centrifugation (4000 rpm, 20 min, 4°C). The pellet was resuspended three times in 15 ml of fresh ASW-AA medium to removed soluble Cd. The CdS precipitates fraction was filtered (F4) before Cd quantification. The samples (F1 to F5) were analyzed by ICP-MS as described in the materials and methods section. The sum of Cd quantities found from fractions F1 to F5 is consistent with the total Cd amount added to the culture (5600 µg, 1 mM Cd in a culture of 50 ml, Cd MW  = 112).
(TIFF)

Figure S3 **Effect of Zn, Ni, Co availability on** ***T. gammatolerans*** **Cd tolerance.** Cd susceptibility was analyzed in ASW-AA-Cyst medium (black line) or in modified ASW-AA-Cyst media supplemented with: 1 mM Cd (yellow line), 1 mM Cd and 200 µM DTPA (metal chelator, blue line); 1 mM Cd and 200 µM DTPA and 100 µM iron (brown line), 100 µM Co (clear pink line), 1 mM Cd and 200 µM DTPA and 100 µM Co (red line), 100 µM Zn (clear purple line), 1 mM Cd and 200 µM DTPA and 100 µM Zn (purple line), 100 µM Ni (clear green line), 1 mM Cd and 200 µM DTPA and 100 µM Ni (green line). The effect of metals availability on Cd tolerance was investigated in each culture medium inoculated with 10^7^ cells/mL and incubated at 85°C on a reciprocal shaker. Cell densities were scored using a Thoma counting chamber. The values are the mean of three independent cultures (SD ≤10%).
(TIFF)

Table S1 **Quantification of cellular Cd by ICP-MS.** The amounts of Cd retained by *T. gammatolerans* cells after a 1 mM Cd challenge were determined in exponentially growing cells for the timepoints 30 min, 120 min, 270 min and 24 hrs (three independent cultures C1, C2, C3 for each timepoint). Following Cd treatment, cells were filtered and pelleted by centrifugation (4000 rpm, 20 min, 4°C). The pellet was resuspended three times in 15 ml of fresh ASW-AA medium to removed soluble Cd. The supernatants were pooled before analysis. The fractions were analyzed by ICP-MS as described in the materials and methods section.
(XLSX)

Table S2 **List of the 114 Cd-responsive genes.** Microarray analyses monitored 161 transcriptional changes (|FC|≥2 and p-Value ≤ 0.01) in response to Cd exposure corresponding to 114 different genes. The expression fold changes and the corresponding p-Value are indicated for each Cd concentration tested (0.1 and 1 mM) and timepoints (30, 120 and 270 min). Up- and downregulated genes are shown in red and green respectively.
(XLS)

Table S3 **List of oligonucleotides used for quantitative real-time RT-PCR.** PCR primer pairs were designed using Primer3 software [79] with standard parameters (product size range: 100–300 bp; optimal primer size: 23b; optimal primer melting temperature: 63°C; optimal primer GC%: 55%) and calibrated with genomic DNA.
(XLSX)
