# Supplementary figures and images for: Genome-Wide Transcriptional Response of the Archaeon Thermococcus gammatolerans to Cadmium
Source: PLoS One. 2012 Jul 27;7(7):e41935. doi: 10.1371/journal.pone.0041935 (PMC3407056; doi:10.1371/journal.pone.0041935)

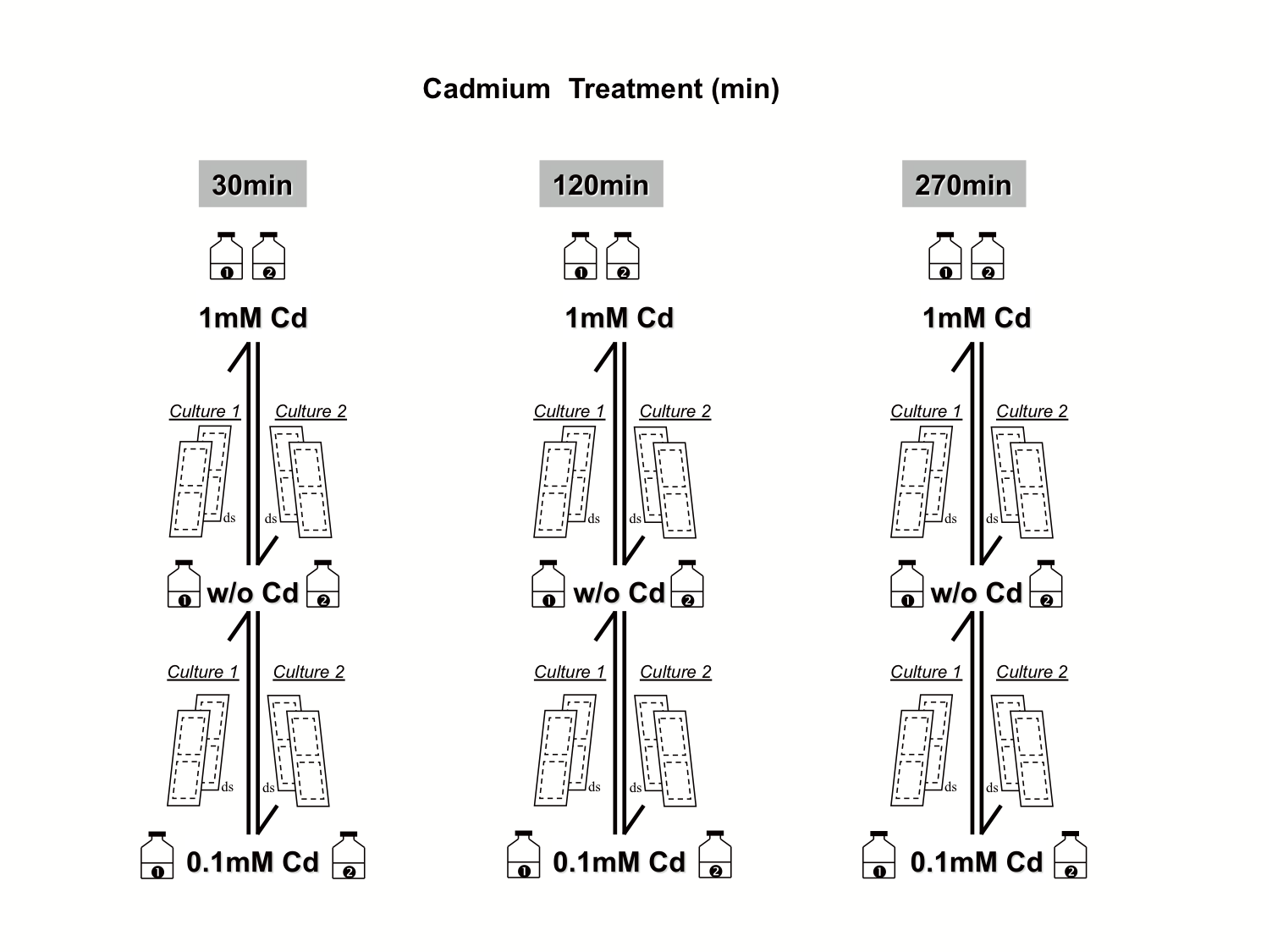

Supplement: Figure S1 — Experimental design of microarray approach. T. gammatolerans exponentially growing cultures were exposed to 0.1 or 1 mM Cd. As a control, cultures without Cd (w/o Cd) were grown in parallel. Time course aliquots were collected 30, 120 and 270 min after Cd exposure. RNA was purified and reverse transcribed. Labeled cDNA were hybridized onto microarrays containing oligonucleotide probes for all ORFs printed in duplicate (GEO accession number GSE13546). The Cd transcriptional response was monitored in two independent cultures for each condition and each biological replication was hybridized twice on a microarray in a dye swap (ds) manner leading to a total of 8 data points per condition and per gene. (TIFF) [file pone.0041935.s001.tiff]

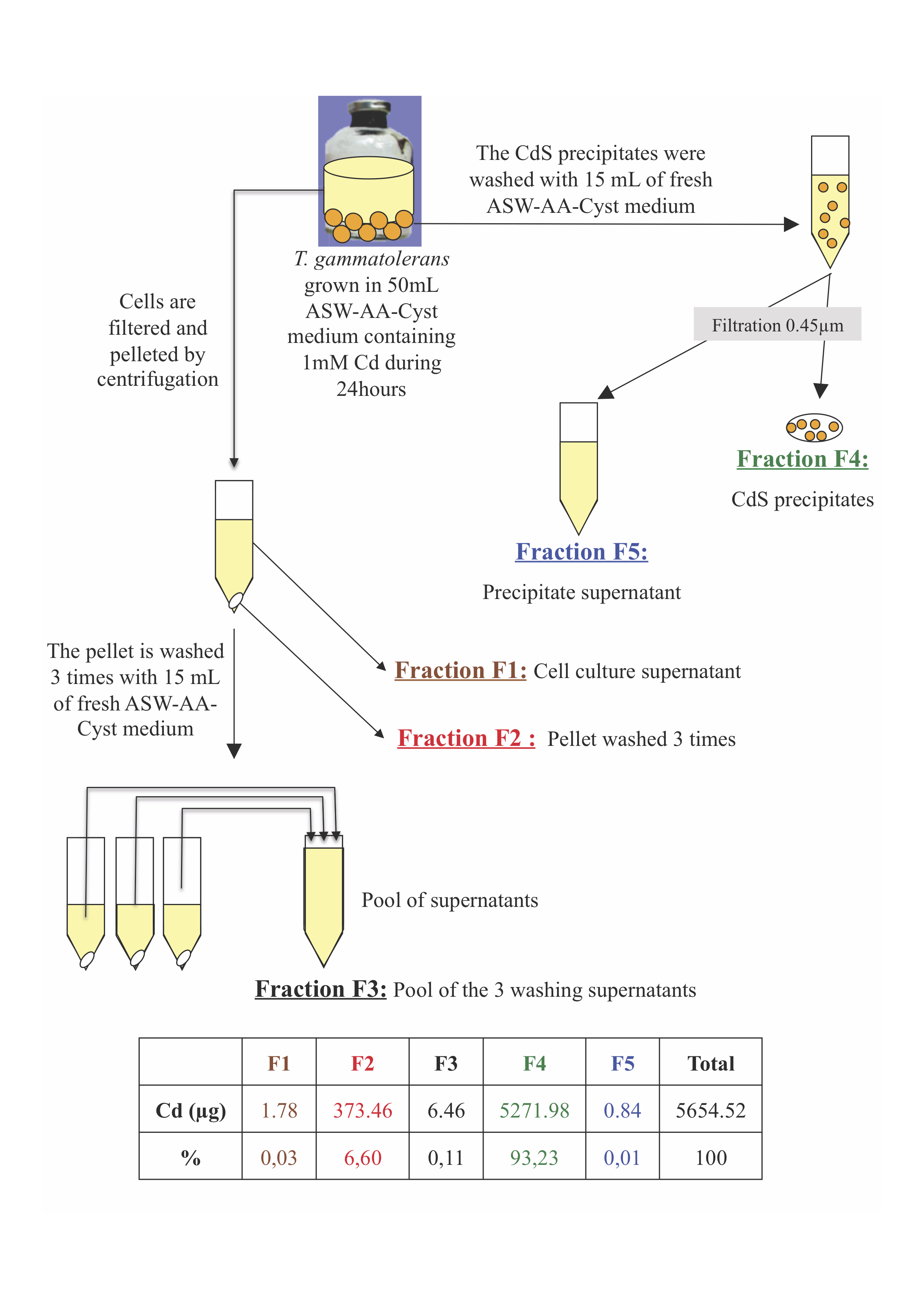

Supplement: Figure S2 — Quantification by ICP-MS of Cd. T. gammatolerans exponentially growing cells (50 mL) were challenged at a cell density of 5×10^7^ cells/mL with 1 mM Cd during 24 hrs. Following Cd treatment, cells were filtered and pelleted by centrifugation (4000 rpm, 20 min, 4°C). The pellet was resuspended three times in 15 ml of fresh ASW-AA medium to removed soluble Cd. The CdS precipitates fraction was filtered (F4) before Cd quantification. The samples (F1 to F5) were analyzed by ICP-MS as described in the materials and methods section. The sum of Cd quantities found from fractions F1 to F5 is consistent with the total Cd amount added to the culture (5600 µg, 1 mM Cd in a culture of 50 ml, Cd MW = 112). (TIFF) [file pone.0041935.s002.tiff]

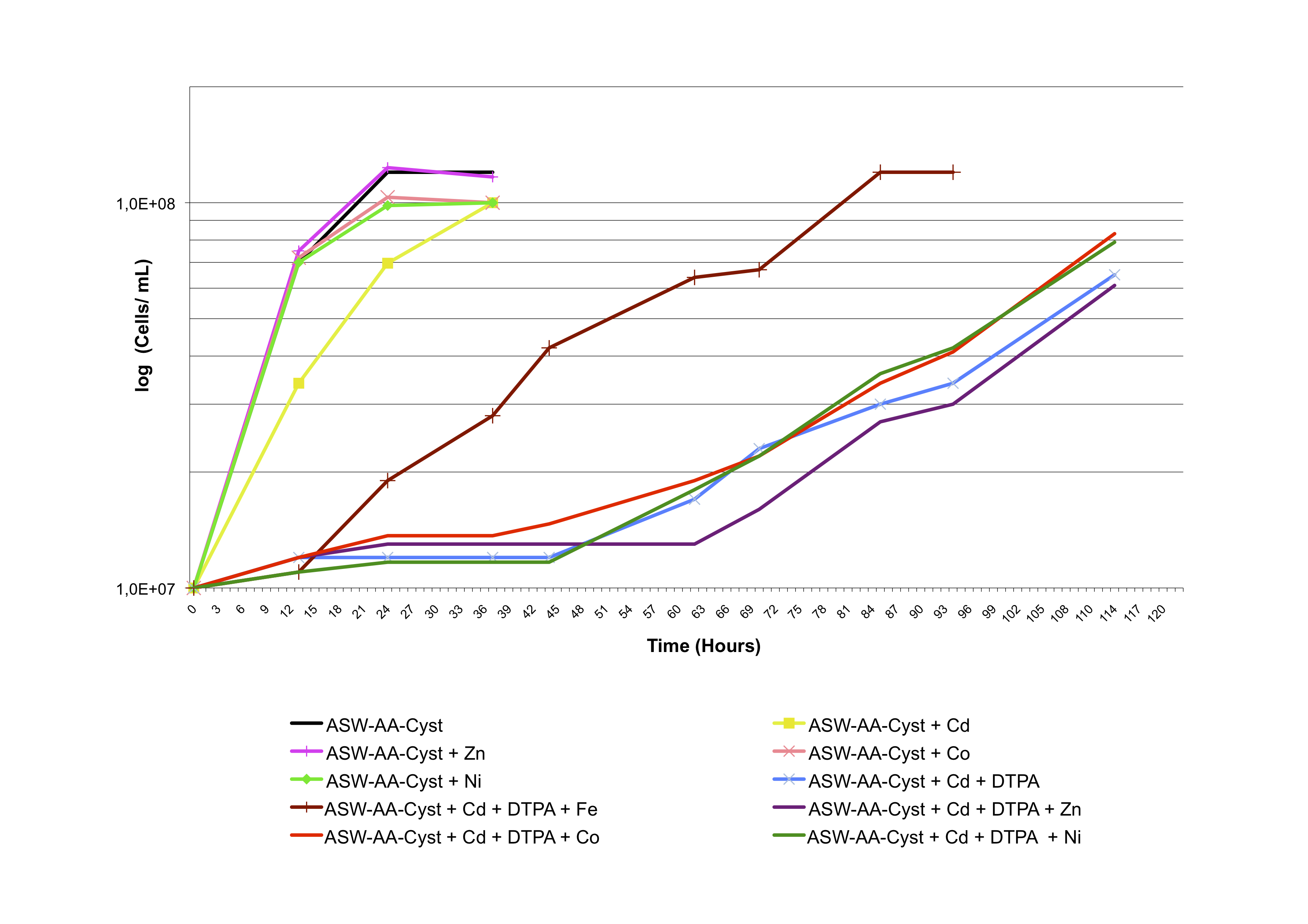

Supplement: Figure S3 — Effect of Zn, Ni, Co availability on T. gammatolerans Cd tolerance. Cd susceptibility was analyzed in ASW-AA-Cyst medium (black line) or in modified ASW-AA-Cyst media supplemented with: 1 mM Cd (yellow line), 1 mM Cd and 200 µM DTPA (metal chelator, blue line); 1 mM Cd and 200 µM DTPA and 100 µM iron (brown line), 100 µM Co (clear pink line), 1 mM Cd and 200 µM DTPA and 100 µM Co (red line), 100 µM Zn (clear purple line), 1 mM Cd and 200 µM DTPA and 100 µM Zn (purple line), 100 µM Ni (clear green line), 1 mM Cd and 200 µM DTPA and 100 µM Ni (green line). The effect of metals availability on Cd tolerance was investigated in each culture medium inoculated with 107 cells/mL and incubated at 85°C on a reciprocal shaker. Cell densities were scored using a Thoma counting chamber. The values are the mean of three independent cultures (SD ≤10%). (TIFF) [file pone.0041935.s003.tiff]
